# Long-Term Thyroid Complications Post-COVID-19: A Systematic Review

**DOI:** 10.3390/microorganisms14030543

**Published:** 2026-02-27

**Authors:** Luai Hommos, Harsh Gohil, Mlaak Rob, Jane Manyama, Haneen Ramy, Nesha Naseem, Hana Nishan, Raghad Sabaawi Ibrahim, Shahad Sabaawi Ibrahim, Vivian Chetachi Eziefula Njoku, Ibrahim Al-Mutawa, Aasiya F. Khan, Sean Holroyd, Dalia Zakaria

**Affiliations:** 1 Department of Medical Education, Weill Cornell Medicine-Qatar, Doha 24144, Qatar; lah4007@qatar-med.cornell.edu (L.H.); hsg4002@qatar-med.cornell.edu (H.G.);; 2 Department of Premedical Education, Weill Cornell Medicine-Qatar, Doha 24144, Qatar

**Keywords:** COVID-19, SARS-CoV-2, post-COVID-19 sequelae, long COVID, thyroid, hypothyroidism, hyperthyroidism, subacute thyroiditis, thyrotoxicosis, NTIS

## Abstract

Coronavirus disease 2019 (COVID-19) is increasingly shown to be a multisystem disorder with long-term complications, including endocrine system complications. The thyroid gland is also susceptible, as it contains ACE2 receptors, making it exposed to both direct viral damage and autoimmune-mediated dysfunction. Recent reports document the various thyroid complications that persist well after the acute infection phase. This systematic review investigates the long-term thyroid complications in individuals with a history of SARS-CoV-2 infection. A comprehensive literature search across several databases was conducted. Eligible studies reported new onset long-term thyroid complications occurring post-COVID-19 infection. Abstract and full-text screening as well as data extraction and quality assessment was performed by two independent reviewers. Only 28 studies met our inclusion criteria, reporting 419 patients from 18 countries. These studies included case reports, case series, cohort, and cross-sectional studies. Reported thyroid disorders included subacute thyroiditis, thyrotoxicosis, hyperthyroidism (including Graves’ disease), isolated high T3/T4, hypothyroidism, central hypothyroidism, and non-thyroidal illness syndrome (NTIS). While many of these eventually resolved, a significant portion persisted or recurred, especially autoimmune thyroiditis. COVID-19 is associated with a range of long-term thyroid complications. Although some cases are temporary, others last, especially autoimmune thyroid disorders. Proposed mechanisms include direct viral cytotoxicity, cytokine-mediated Hypothalamic–Pituitary–Thyroid (HPT) axis suppression, post-viral autoimmunity, vascular injury, and neuroendocrine disruption. Routine thyroid function monitoring in COVID-19 survivors, particularly those with severe disease or persistent symptoms is recommended, and larger prospective studies are needed to better understand incidence and outcomes.

## 1. Introduction

Coronavirus disease 2019 (COVID-19), caused by the Severe Acute Respiratory Syndrome Coronavirus 2 (SARS-CoV-2), was first identified in late 2019 and then quickly escalated into a global pandemic [1]. While initially characterized as a viral respiratory illness, it has become increasingly clear that COVID-19 as a disease affects multiple organ systems and has long-term health consequences that extend far past the lungs [2]. This multisystem effect of COVID-19 is largely attributable to the widespread distribution of angiotensin-converting enzyme 2 (ACE2) receptors, among others, which the virus uses for cell entry [3]. These receptors are expressed not only in the respiratory tract but also in the vascular endothelium, gastrointestinal system, kidneys, nervous tissue, and endocrine glands, including the thyroid [4]. This leads to many organ-specific clinical symptoms like neurological symptoms such as anosmia and encephalopathy, cardiovascular complications such as myocarditis and arrhythmias, as well as other complications like gastrointestinal disturbances and endocrine abnormalities [5]. Moreover, COVID-19-induced organ damage may necessitate solid organ transplantation [6].

Further, an estimated 10% of people experience long-term symptoms, referred to as “long COVID” [7]. These may persist for weeks or months after the resolution of acute illness and include fatigue, dyspnea, palpitations, neurocognitive impairment (also called “brain fog”), and endocrine dysfunction [8]. Specific organ systems, including gastrointestinal and hepatic systems, are affected in long COVID. Manifestations in these systems include steatosis, non-alcoholic fatty liver disease (NAFLD), fibrosis, cirrhosis, hepatitis, and even acute liver failure [9,10]. Endocrine sequelae encompassing new-onset diabetes, predominantly type 2 but also type 1 are also well documented among long COVID patients [11]. Notably, emerging data suggest that COVID-19 may have a lasting impact on thyroid function, with some studies showing the prevalence of autoimmune thyroid diseases doubling in COVID-19 survivors [12]. In severe cases, COVID-19 is marked by a dysregulated host immune response, known as a “cytokine storm”. This hyperinflammatory state is characterized by the excessive release of pro-inflammatory cytokines, such as interleukin-6 (IL-6), tumor necrosis factor-alpha (TNF-α), and interleukin-1 beta (IL-1β) [13]. This can result in vascular leakage, thrombosis, multiorgan failure, and even increased mortality [13]. The cytokine storm is recognized as a major contributor to systemic complications in critically ill patients.

COVID-19 has also been implicated in autoimmune diseases, and it may be a trigger for autoimmune illnesses. Several mechanisms have been proposed to explain how the SARS-CoV-2 virus may disrupt immune tolerance and trigger autoimmunity. One such mechanism is molecular mimicry, whereby viral peptides share structural similarities with host proteins. In one study by Gammazza et al. [14], the researchers compared SARS-CoV-2 viral proteins with human molecular chaperones, particularly heat shock proteins, to identify shared amino acid sequences. Their findings suggest that these shared sequences could lead to cross-reactive immune responses, where the body’s defense mechanisms against the virus might accidentally target its own proteins, potentially triggering autoimmune conditions [14]. Another mechanism is bystander activation, which is the non-specific activation of autoreactive T lymphocytes during times of heightened immune responses. In the setting of COVID-19, particularly during a cytokine storm, elevated levels of pro-inflammatory cytokines, such as those that have been previously mentioned, can lower the activation threshold for T cells. This environment can lead to the inappropriate activation of self-reactive T cells that were previously under regulatory control, leading to the autoimmunity seen in this mechanism [15]. A third proposed mechanism is epitope spreading, which occurs when virus-induced tissue injury leads to the release of antigens that were previously hidden from the immune system. In a study by Shrock et al. [16], the researchers conducted an analysis of antibody responses in COVID-19 patients and found that people with severe disease exhibited a broader and more diverse antibody response, targeting not only SARS-CoV-2 antigens but also antigens from other common human coronaviruses. This broader immune response suggests that, in severe cases, the immune system may expand its reactivity beyond the initial viral epitopes. This potentially leads to the recognition of self-antigens due to the combined effect of epitope spreading and molecular mimicry [16]. These mechanisms help explain clinical reports of COVID-19-associated autoimmune diseases, including Guillain–Barré syndrome, systemic lupus erythematosus, Type 1 diabetes mellitus, and autoimmune thyroid diseases such as Hashimoto’s thyroiditis and Graves’ disease [17]. Epidemiological studies have further noted a rise in autoimmune diagnoses during the pandemic [18].

Among the endocrine organs, the thyroid gland appears to be specifically susceptible to dysfunction during and after COVID-19 infection, with acute and long-term complications. Several forms of thyroid dysfunction have been reported in relation to COVID-19. These include subacute thyroiditis (SAT) and painless thyroiditis, and in hospitalized patients with severe COVID-19, non-thyroidal illness syndrome (NTIS) is also commonly observed and is thought to perhaps reflect an adaptive metabolic response to critical illness [19,20]. Additionally, cases of new-onset autoimmune thyroid diseases, such as Graves’ disease and Hashimoto’s thyroiditis, have been reported following SARS-CoV-2 infection, with some patients experiencing relapses of previously well-controlled autoimmune thyroid disorders [21,22]. These findings suggest that both direct viral effects and immune-mediated mechanisms may contribute to thyroid involvement in COVID-19. While cases recorded of thyroid dysfunction in COVID-19 are often transient, a subset of patients may develop persistent thyroid disease. In a prospective cohort, thyroid hormone abnormalities resolved in most patients within 3 months of recovery, but some developed new autoimmune thyroiditis, indicating a potential for the long-term risk of autoimmune diseases [23].

Many studies have highlighted the onset of thyroid disorders following COVID-19 infection, both as a transient complication and permanent sequelae. In light of these observations, we aim to compile the reported long-term thyroid complications in individuals with a history of SARS-CoV-2 infection. The study included cohort-based analyses as well as case reports, as both offer valuable insights into the possible long-term thyroid complications associated with SARS-CoV-2 infection. Including both cohort studies and case reports allowed for a more comprehensive synthesis of the available evidence. Case reports complemented cohort data by including rare presentations, early signals, methods of diagnosis, and management. 

## 2. Materials and Methods

The protocol of this systematic review (registration number: INPLASY2025110091) was developed according to the Preferred Reporting Items for Systematic Reviews and Meta-Analyses (PRISMA) [24].

### 2.1. Information Sources and Search Strategy

This study is part of a comprehensive project looking at the long-term and severe complications of COVID-19. A comprehensive search was conducted by an information professional. The search strategy was designed to maximize sensitivity in order to retrieve all relevant studies. The following databases were searched in October 2023: PubMed, Medline (Ovid, 1946–Current), Embase (Ovid, 1974–2021), Scopus, Web of Science, Science Direct, and Cochrane Library. Keywords and controlled vocabulary that focused on “Long Covid” and variants were used with no language or date restrictions (Supplementary File S1). All database search results were imported into EndNote (version 19) and exported to Covidence, where duplicates were removed prior to the initial screening.

### 2.2. Eligibility Criteria

Articles without primary data, such as review articles, were excluded, with no restrictions based on country, age, or gender. Only full articles that were in English were included, and any conference abstracts were excluded. During the full text screening, any studies that reported long-term thyroid complications post-COVID-19 were included. This includes patients who developed thyroid complications after recovering from COVID-19. If the disorder was diagnosed after at least a month after COVID-19 diagnosis or if the study reports that anti-SARS-CoV-2 IgG but not IgM antibodies were detected, the study was included. Studies reporting related diagnosis during the active COVID-19 infection were included only if the symptoms lasted for more than 12 weeks after COVID-19 diagnosis, if the patients received a treatment for the disorder for at least 12 weeks after COVID-19 diagnosis, or if the patient died before 12 weeks. Any thyroid conditions that were diagnosed during the active infection of COVID-19 and fully recovered within less than 12 weeks and those with a history of thyroid illness were excluded.

### 2.3. Study Selection and Data Collection

Screening, including title and abstract and full-text screening, as well as data extraction, was conducted by two independent reviewers for each study using Covidence. Any disagreements were resolved by consensus.

### 2.4. Data Items

Demographic and clinical data, including age, sex, comorbidities, treatments, and outcomes, were collected. Continuous variables were expressed as the mean ± standard error or range of results. Categorical variables were expressed as percentages.

### 2.5. Risk of Bias and Quality Assessment

The quality of the included studies was assessed using different methods, depending on the type of study. The Newcastle–Ottawa Quality Assessment Scale was used to assess the cohort studies [25], and the scale developed by Murad et al. [26] was used to assess the case reports and case series. Quality assessment was conducted by two independent reviewers.

### 2.6. Data Analysis

The thyroid conditions reported by the included studies were classified, and the numbers of patients were compiled from all studies under each category.

## 3. Results

**Figure 1:**
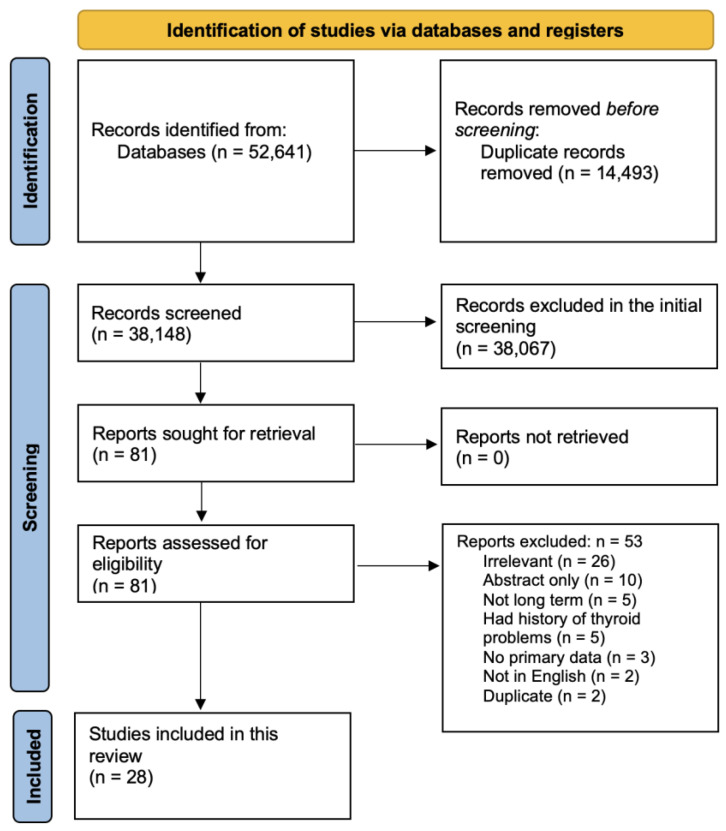
Screening and study selection protocol.

Figure 1 shows the flow diagram of the study protocol. After removing the duplicates, the titles and abstracts of 38,148 studies were screened, of which 81 were selected for full text screening. Only 28 studies met our inclusion criteria. Of the 53 excluded studies, 26 were irrelevant, 3 had no primary data, 10 were not peer reviewed or were abstracts only, 2 were not in English, 2 were duplicates, 5 were not classified as long-term complications, and 5 had a history of thyroid complications. Supplementary Table S1 summarizes the demographic and clinical data of the included subjects as well as the quality assessment score for each study [27,28,29,30,31,32,33,34,35,36,37,38,39,40,41,42,43,44,45,46,47,48,49,50,51,52,53,54].

### 3.1. Types of Studies and Demographic Data

Of the 28 included studies, 13 were case reports, 1 was a case series, 10 were cohort studies, 2 were case–control studies, and 2 were cross-sectional studies. Among the 28 studies, 5 were from the USA, 3 from Italy, 2 each from India, Turkey, Hungary, and Iraq, and 1 each from the UAE, China, Korea, Qatar, Russia, Iran, Bosnia and Herzegovina, Pakistan, Vietnam, Hong Kong, and Bulgaria, with 1 from Hong Kong and China together.

The total number of COVID-19 patients reported by the included studies was 408,785 of whom 51.46% were males. The studies also reported 2,037,581 control patients (Supplementary Table S1).

### 3.2. Clinical Data

**Figure 2:**
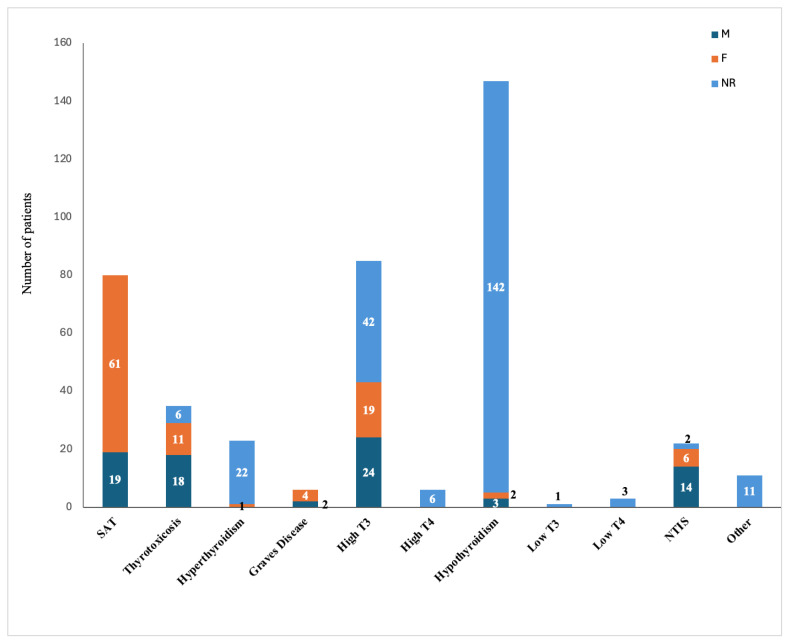
Types of thyroid complications post-COVID-19 infection. F: female, M: male, NR: not reported, NTIS: Non-Thyroidal Illness Syndrome, SAT: Subacute Thyroiditis, T3: Triiodothyronine, T4: Thyroxine.

The 28 included studies reported a total of 419 patients with thyroid disorders post-COVID-19, of whom 24.8% were females, 19.1% were males, and the gender was not reported for the remaining 56.1%. Table 1 and Figure 2 show the types of reported thyroid conditions post-COVID-19, which were SAT, thyrotoxicosis, hyperthyroidism, Graves’ disease, high triiodothyronine (T3), high thyroxine (T4), hypothyroidism, low T3, low T4, and NTIS.

## 4. Discussion

This systematic review included 28 studies from 18 different countries reporting 419 patients who developed thyroid complications post-COVID-19 infection. The included studies reported a wide range of thyroid disorders including 235 cases with elevated thyroid hormones, 173 with low thyroid hormones, and 11 with unspecified conditions (3 with high Thyroid-Stimulating Hormone (TSH) and 8 with unspecified autoimmune thyroiditis).

### 4.1. Disorders Including High Thyroid Hormones

**Figure 3:**
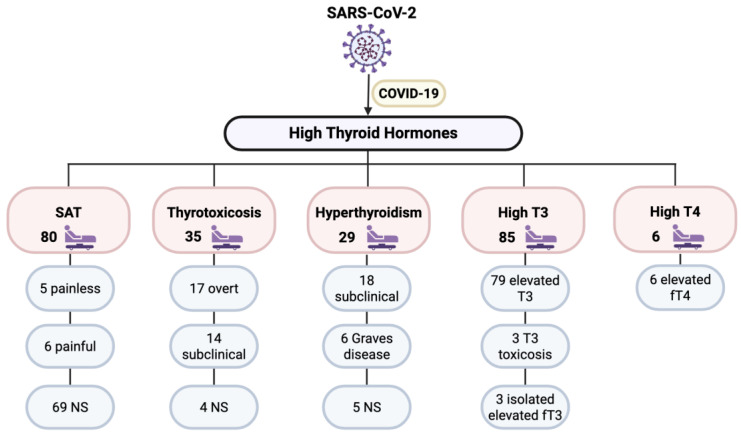
Disorders including high thyroid hormones. fT3: free T3, fT4: free T4, NS: not specified, SAT: Subacute Thyroiditis, T3: Triiodothyronine, T4: Thyroxine.

Our included studies reported 235 cases of elevated thyroid hormones classified as SAT, thyrotoxicosis, hyperthyroidism (including Graves’ disease), high T3, or high T4. Figure 3 summarizes the types of post-COVID-19 thyroid complications that included high thyroid hormones as reported by the included studies.

#### 4.1.1. Subacute Thyroiditis

SAT, also known as de Quervain’s thyroiditis or subacute granulomatous thyroiditis, is a self-limited inflammatory condition of the thyroid gland. Painful SAT (PFSAT) is characterized by neck pain or tenderness, often radiating to the jaw or ear, and symptoms of thyrotoxicosis. It is the most common cause of thyroid pain, which develops with an initial thyrotoxic phase lasting 3–6 weeks, followed by a transient hypothyroid phase that can persist for up to 6 months, and eventual resolution to euthyroidism within 12 months [55,56]. In contrast, painless SAT (PLSAT), also known as silent thyroiditis or subacute lymphocytic thyroiditis, shares the same triphasic course but lacks thyroid pain and is more commonly associated with autoimmune mechanisms [57]. Both forms of SAT result from the unregulated release of preformed thyroid hormones from damaged thyroid follicular cells during the thyrotoxic phase, which is often followed by hypothyroidism, as the hormone stores are depleted [56,57]. Although SAT is generally self-limited, up to 5–15% of patients may develop permanent hypothyroidism [57]. Understanding the clinical course and distinguishing features of painful and painless SAT is essential for accurate diagnosis and effective management.

Across the included studies, a total of 80 cases of SAT were reported following COVID-19 infection, with 5 cases classified as PLSAT, 6 as PFSAT, and the remainder unspecified. Cohort-based analyses offered valuable insight into the possible long-term thyroid complications associated with SARS-CoV-2 infection. For example, Lee et al. [31] compared SAT incidence between COVID-19 survivors and the general population. SAT occurred more frequently in females, possibly due to sex-based immune differences. The study reported a SAT incident rate (IR) per 100,000 persons of 17.28 (95% confidence interval [CI], 12.56–23.20) in the COVID-19 group and 8.63 (95% CI, 6.37–11.45) in the control group with an HR of 1.93 (95% CI, 1.43 to 2.59; *p* < 0.01). Furthermore, the cumulative IR of SAT was higher in the COVID-19 group compared to the control group over a 2-year period, and the HR for COVID-19 increased to 2.30 (95% CI, 1.60–3.30), *p* < 0.001) after 180 days. The study also investigated the effect of severity of COVID-19 and reported that the HR in hospitalized patients for COVID-19 was 2.00 (95% CI, 1.41–2.83) (*p* < 0.001) compared with the outpatient clinic with an HR of 1.76 (95% CI, 1.01–3.06).

Similarly, Mondal et al. [47] identified a 6.8% SAT incidence within 3 months of COVID-19 recovery. The analysis distinguished between painless (*n* = 5) and painful (*n* = 6) SAT cases, finding that PLSAT patients presented earlier, exhibited more severe thyrotoxic symptoms, and had higher C-reactive protein (CRP), IL-6, and neutrophil–lymphocyte ratios alongside lower lymphocyte counts. Treatment evaluation highlighted the role of oral glucocorticoids in symptom control, with the six-month follow-up revealing gradual clinical and biochemical recovery.

Other cohort and case series studies further support the link between COVID-19 and SAT. Elhadd et al. [32] described 10 cases of thyroid dysfunction in Qatar, including one SAT case, and proposed that immune hyperactivity and cytokine release syndrome, mechanisms also implicated in autoimmune thyroid disease [58,59], may underlie post-COVID thyroid injury. They also noted a possible bimodal pattern of thyroid dysfunction, occurring during both acute infection and the recovery period, with comorbidities such as asthma, hypertension, and diabetes potentially complicating management. These findings collectively underscore the importance of thyroid screening in post-COVID populations.

Individual case reports further illustrate the clinical spectrum, timing, and management strategies of SAT after COVID-19. Ragab [48] described a previously healthy 40-year-old male who developed SAT 5 months after asymptomatic infection, presenting with localized neck pain and sore throat. Ultrasound revealed an enlarged thyroid lobe with inhomogeneous margins, and histopathology indicated granulomatous thyroiditis. The patient recovered fully with nonsteroidal anti-inflammatory drugs. In contrast, Al-Shammaa and Abdlkadir [27] reported a 53-year-old female presenting with neck tenderness and tachycardia 10 days post-COVID recovery; low radioactive iodine uptake confirmed SAT, and the patient improved with beta-blockers and analgesics, later requiring prednisone for symptom resolution. Likewise, Mehmoud et al. [28] described a 29-year-old woman who developed progressive SAT symptoms 10 days after COVID-19 recovery, including dysphagia, weight loss, and fever. Laboratory evaluation revealed marked thyrotoxicosis with elevated inflammatory markers; high-dose prednisone and atenolol led to improvement.

Mechanistically, these reports align with current hypotheses that SARS-CoV-2 can bind to ACE2 receptors expressed in thyroid follicular cells [60], facilitating direct viral injury. Additionally, the inflammatory response in COVID-19 may recruit cytotoxic T cells and macrophages to thyroid tissue, amplifying follicular cell damage and contributing to the clinical and biochemical profiles observed in SAT [61].

#### 4.1.2. Thyrotoxicosis

Thyrotoxicosis is a clinical disorder caused by excess levels of thyroid hormones that cause the increased activity of primarily T3 and T4 [62,63]. There is a wide range of clinical presentations from asymptomatic to life-threatening. The symptoms associated with this disorder include severe weight loss and palpitations. However, it may also present as a severe disorder with complications such as heart failure, atrial fibrillation, and, in extreme cases, death [63].

Our included studies reported 35 patients with thyrotoxicosis post-COVID-19, which developed either during the active infection or after recovery. For example, Lui et al. [39] reported 204 COVID-19 survivors, of whom, 43 patients had abnormal thyroid function tests (TFTs), including 13 patients who were diagnosed with subclinical thyrotoxicosis during the active infection. They were reassessed approximately 3 months post-acute infection, and three patients persisted in subclinical thyrotoxicosis.

The prospective study conducted by Pizzocaro et al. [41] offers perspective on how thyrotoxicosis caused by COVID-19 progresses over time. The study followed 29 patients who developed COVID-19-related thyrotoxicosis. The patients included had one of two forms: subclinical or overt thyrotoxicosis. At follow-up, 28 patients developed euthyroid status, while one patient developed overt hypothyroidism. Ultrasound was an important aspect of this study, as it was found that 10 patients had hypoecogenicity of the thyroid gland, and those patients tend to have higher TSH levels compared to patients who had no hypoecogenicity.

COVID-19-related thyrotoxicosis may often be secondary to destructive thyroiditis. Histopathological evidence supports this, showing apoptotic thyroid cells with minimal lymphocytic infiltration and the absence of thyrotropin receptor antibodies, which rules out Graves’ disease in some cases [64,65]. The high expression of ACE2 in thyroid tissue supports viral entry as a trigger for this inflammatory damage.

#### 4.1.3. Hyperthyroidism

Hyperthyroidism, which is characterized by the excess production of thyroid hormones, can lead to a range of symptoms including weight loss, increased heart rate, anxiety, and tremors. While hyperthyroidism is commonly caused by conditions like Graves’ disease, recent studies have suggested that COVID-19 infection may also play a role in triggering thyroid dysfunction, including hyperthyroidism.

Hyperthyroidism has been reported after COVID-19 in both subclinical and overt forms, with our included studies identifying 23 post-infection cases. The condition often appears in the subacute recovery phase, suggesting that COVID-19 may act as a trigger for thyroid hyperfunction through immune activation and post-viral inflammatory processes.

Some patients, such as those described by Ilyaev [35] and Seres et al. [45], developed symptomatic thyrotoxicosis after COVID-19, with tremors, weight loss, anxiety, and tachycardia, accompanied by suppressed TSH and elevated thyroid hormones. In certain cases, like that reported by Whiting et al. [46], post-COVID thyroid inflammation led initially to hypothyroid-pattern labs. However, it was acknowledged as capable of producing a transient hyperthyroid phase through unregulated hormone release from damaged follicles. Semenova [54] identified subclinical hyperthyroidism in 12.3% of COVID-19 patients, reinforcing the possibility of a mild often self-limiting form in some individuals. While many cases resolve spontaneously or with symptomatic treatment, a subset progresses to persistent hyperthyroidism or reveals underlying autoimmune disease.

Graves’ disease, a well-recognized autoimmune cause of hyperthyroidism, was reported in six post-COVID cases across five studies. The pathogenesis involves thyroid-stimulating immunoglobulins binding to the TSH receptor, causing sustained hormone synthesis and glandular enlargement [66]. COVID-19-associated immune dysregulation may precipitate Graves’ disease via molecular mimicry, enhanced B-cell activation, and cytokine-driven inflammation [67,68]. Elhadd et al. [32] documented five Graves’ cases in a series of 10 post-COVID thyroid dysfunction patients, noting a bimodal distribution of onset during acute infection and recovery. Rarely, COVID-19 appears to trigger multiple autoimmune conditions simultaneously, as in De Giglio et al. [44], where a patient developed both Graves’ disease and myasthenia gravis after infection. Additional reports, such as Feghali et al. [40], describe Graves’ disease arising alongside other post-COVID autoimmune thyroiditis presentations, further supporting an immune-mediated mechanism.

Collectively, these findings suggest that hyperthyroidism after COVID-19 can manifest through two primary pathways: a transient destructive thyroiditis resulting in short-lived thyrotoxicosis or an autoimmune process leading to persistent disease such as Graves’ hyperthyroidism. The latter warrants ongoing surveillance, as its onset may be delayed for weeks to months after the acute infection. Given the variability in presentation and outcomes, post-COVID patients, especially those with compatible symptoms, should be screened for thyroid dysfunction to ensure timely diagnosis and management.

#### 4.1.4. High T3 or High T4

The included studies reported 85 patients with high T3 and 6 with high T4. The conditions were either described as isolated elevated T3 or T4 or as high T3 or T4, without clarifying the other factors leading to a diagnosis. For example, Malik et al. [52] compared the thyroid hormone levels of COVID-19 patients to a matched group of non-COVID-19 patients. The findings showed that patients with COVID-19 pneumonia had significantly higher TSH levels and significantly lower T3 levels in comparison to non-COVID-19 pneumonia of similar severity. This conclusion led to the suggestion that COVID-19 has a significant impact on thyroid function and acute phase reactants, regardless of how mild or severe the infection was. It must also be noted that sick euthyroidism was not observed in the COVID-19 cohort. Semenova et al. [54] evaluated the thyroid function and TNF-α levels in 85 post-reproductive women aged 45–69 during the acute phase of moderate COVID-19 and 12 months later. Participants were divided into control, acute COVID-19, 12-months post-COVID-19, and asymptomatic COVID-19 groups. It was found that 75.4% of COVID-19 patients had euthyroidism, while 12.3% had subclinical hyperthyroidism. The fT4 levels were significantly higher in women with COVID-19 compared to controls and asymptomatic patients. The study suggests COVID-19 may trigger thyroid dysfunction through inflammatory responses involving TNF-α, as indicated by the positive correlation between fT4 and TNF-α levels (r = 0.38; *p* = 0.004) [69,70]. The authors concluded that moderate COVID-19 in post-reproductive women is linked to increased fT4 levels, which persist for at least 12 months. Long-term thyroid monitoring in these women is recommended to diagnose potential thyroid conditions early. The study highlighted that post-reproductive women are particularly vulnerable to thyroid dysfunction following COVID-19 due to age-related estrogen deficiency, which can exacerbate thyroid and other organ disorders.

### 4.2. Disorders Including Low Thyroid Hormones

**Figure 4:**
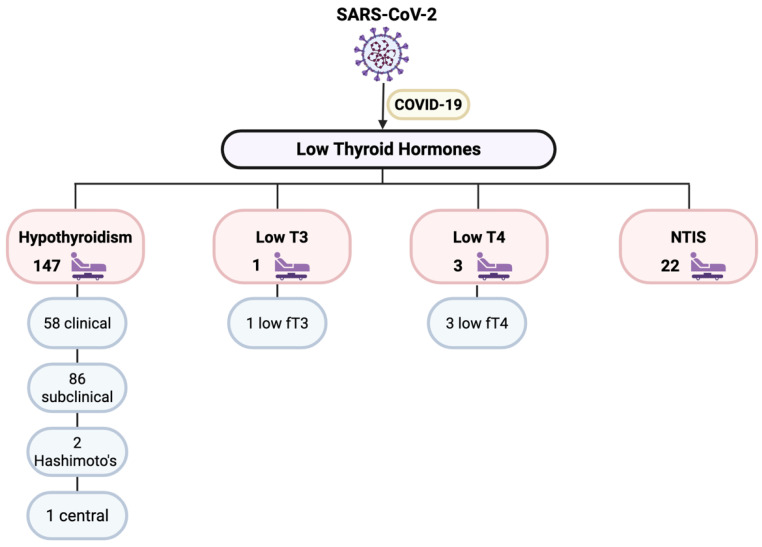
Disorders including low thyroid hormones. fT3: free T3, fT4: free T4, NTIS: Non-Thyroidal Illness Syndrome, T3: Triiodothyronine, T4: Thyroxine.

Our included studies reported 173 cases of low thyroid hormones classified as hypothyroidism (including Hashimoto’s disease), NTIS, low T3, or low T4. Figure 4 summarizes the types of post-COVID thyroid complications that included low thyroid hormones as reported by the included studies.

#### 4.2.1. Hypothyroidism

Hypothyroidism, a condition which involves an underproduction of thyroid hormones, can be divided into overt hypothyroidism (clinical hypothyroidism) and subclinical hypothyroidism (SCH). Overt hypothyroidism presents with elevated TSH and low T3/T4; subclinical hypothyroidism presents with elevated TSH but normal T3/T4 [71]. While autoimmune diseases, like Hashimoto’s thyroiditis, are seen as the most common cause of hypothyroidism, viral infections, including COVID-19, have also been considered relevant in the pathogenesis of thyroid dysfunction.

Across the included literature, 147 post-infection cases of hypothyroidism were identified, suggesting that SARS-CoV-2 may have a lasting impact on thyroid function. Onset is typically delayed, occurring weeks to months after recovery, and can present with a range of severities from subtle biochemical changes to profound hormone deficiency. The mechanisms appear multifactorial, with evidence for direct viral effects, post-viral immune dysregulation, and secondary thyroid damage from systemic inflammation.

Several case reports illustrate the pattern of delayed-onset hypothyroidism. Burekovic et al. [36] described a 38-year-old patient who developed hypothyroidism six weeks after recovery, while Trinh [50] reported a similar presentation in a 28-year-old woman at eight weeks post-infection. Both cases highlight the potential for new-onset disease in previously healthy individuals. Larger cohort studies support these observations. Adhikari and Singh [72] found SCH in 60.53% of patients within 3 months of recovery, characterized by elevated TSH with normal T3 and T4 levels and persisting in some patients beyond the acute phase. Alphan Uc [49] also documented high rates of SCH and NTIS in hospitalized COVID-19 patients, particularly in severe cases. While most returned to euthyroid status within six months, a proportion developed persistent SCH or new anti-thyroid antibody positivity, suggesting a post-infectious autoimmune process.

The autoimmune link is reinforced by cases of Hashimoto’s thyroiditis following COVID-19. Feghali et al. [40] reported a 38-year-old healthcare worker who developed classic hypothyroid symptoms and marked thyroid enlargement six weeks after infection. Laboratory testing revealed severely elevated TSH, low fT4, and high titers of anti-Thyroid Peroxidase (anti-TPO) and anti-thyroglobulin antibodies; histology confirmed autoimmune thyroiditis. Thyroid hormone replacement led to full symptom resolution. Similar cases without prior thyroid history point toward SARS-CoV-2-induced immune activation potentially via molecular mimicry, bystander activation, or epitope spreading as a trigger for autoimmune disease.

Population-level studies further emphasize the association between COVID-19 severity, inflammation, and thyroid dysfunction. In the Alphan Uc et al. [49] cohort of 163 hospitalized patients, more than half had thyroid abnormalities on admission, most commonly NTIS. Anti-thyroid antibodies were present in 8% (anti-Tg) and 15.3% (anti-TPO) at baseline, with new antibody positivity developing in some at six months. Higher inflammatory markers and lower lymphocyte counts correlated with thyroid dysfunction, and severe cases showed significantly lower T3 levels alongside higher rates of intensive care admission and mortality.

Pediatric and post-inflammatory syndromes also appear to play a role. Garai et al. [33] found that 12% of children with long-COVID symptoms had thyroid autoantibodies, and 7% were diagnosed with autoimmune thyroiditis by ultrasound. Similarly, Bhatt et al. [43] described a case of autoimmune thyroiditis within the context of multisystem inflammatory syndrome in adults (MIS-A), underscoring that post-infectious immune activation may target the thyroid as part of a broader inflammatory response.

Taken together, the evidence indicates that COVID-19 related hypothyroidism most often arises from autoimmune thyroiditis, either de novo or as an exacerbation of pre-existing subclinical disease. Cytokine-mediated follicular damage, sustained inflammation, and the emergence of thyroid autoantibodies are central to its pathogenesis. While many patients recover, a significant subset develop persistent SCH or overt hypothyroidism, highlighting the need for long-term thyroid function monitoring in COVID-19 survivors, particularly those with severe infection or elevated inflammatory markers during the acute phase.

#### 4.2.2. Central Hypothyroidism

Central hypothyroidism (CH) is a rare form of hypothyroidism caused by insufficient stimulation of an otherwise normal thyroid gland by TSH. It arises from defects in either the pituitary gland (secondary hypothyroidism) or the hypothalamus (tertiary hypothyroidism) or both, leading to reduced thyroid hormone production. CH is approximately 1000 times rarer than primary hypothyroidism and is often associated with low or normal TSH levels, in contrast to the elevated TSH levels seen in primary hypothyroidism [73,74]. The diagnosis of CH is typically suggested by the presence of low thyroid hormone concentrations alongside inappropriately low or normal TSH levels. While thyroid hormone replacement therapy is the cornerstone of treatment, managing CH is more complex compared to primary hypothyroidism, as circulating TSH levels have limited diagnostic value in this condition. Furthermore, CH frequently coexists with other pituitary deficiencies, complicating the clinical management and requiring additional hormone replacement [74].

Elhadd et al. [32] presented 10 cases of thyroid dysfunction in Qatar, including one instance of central hypothyroidism following COVID-19 infection. The study suggests that COVID-19 may contribute to thyroid abnormalities through immune-mediated mechanisms such as heightened cytokine release and immune system activation—processes similarly observed in other thyroid-related conditions [58,75]. The researchers stress the necessity of vigilant thyroid function monitoring in individuals recovering from COVID-19 to prevent underdiagnosis. Their findings support a possible biphasic pattern of thyroid dysfunction associated with the virus, manifesting both during active infection and after recovery. They also note that coexisting health conditions like asthma, hypertension, and diabetes may further complicate the clinical course of thyroid disorders post-COVID-19 [32].

SARS-CoV-2 can impair the central regulation of thyroid function by damaging the hypothalamus and pituitary gland, which express ACE2 and TMPRSS2, facilitating viral entry [76]. Post-infection, CH may manifest due to impaired TSH secretion. Cytokines, particularly IL-6, can also disrupt hormonal feedback in the hypothalamic–pituitary–thyroid (HPT) axis [77], highlighting a multifactorial etiology.

#### 4.2.3. Low T3/T4 and NTIS

Several studies reported cases with reduced T3 or T4 without specifying a diagnosis, while others explicitly identified non-thyroidal illness syndrome (NTIS). NTIS, also known as euthyroid sick syndrome or low T3 syndrome, is typically observed in either starved or critically ill patients. It is characterized by a low serum T3 and is sometimes accompanied by a low T4 and elevated levels of reverse T3 (rT3). TSH levels are usually normal or low, reflecting an inadequate response of the hypothalamic–pituitary–thyroid axis to reduced T3 [78,79]. There is no established consensus on the treatment for this condition, and the severity of NTIS might be linked to worse outcomes. A study by Croce et al. [53] explored the thyroid function among COVID-19 patients who were eligible for respiratory rehabilitation and monitored their thyroid function over time. The study examined the thyroid function of 39 post-COVID inpatients (rehabilitation group) and outpatients (control group) to analyze changes in the thyroid function, specifically, fT3, TSH, and fT4 levels, over 3 months. Initially, the rehabilitation group (20 patients) had lower fT3 levels and went through more intensive respiratory support than the control group (19 patients). Patients in the rehabilitation group showed a significant increase in fT3 levels after 3 months, correlated with improved lower extremity strength and exercise tolerance, as evidenced by better Six-Minute Walking Test (6MWT) and short physical performance battery test (SPPB) results. The study concludes that pulmonary rehabilitation significantly improves fT3 levels and functional capabilities in post-COVID-19 patients, suggesting it helps reverse NTIS.

COVID-19 may induce NTIS, which involves reduced serum T3 and sometimes TSH, without intrinsic thyroid disease. This may occur by suppressing thyrotropin-releasing hormone (TRH) and TSH release and decrease deiodinase activity, particularly D1, impairing peripheral conversion of T4 to T3 [80]. This condition reflects a systemic adaptation to severe illness but can complicate post-COVID thyroid function assessments.

### 4.3. Possible Mechanisms of COVID-19-Induced Thyroid Disorders

**Figure 5:**
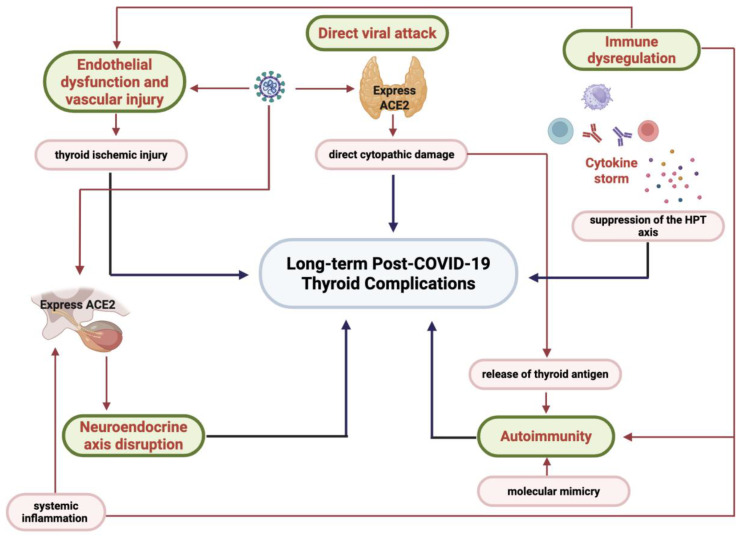
Possible mechanisms of COVID-19–induced thyroid disorders. ACE2: Angiotensin Converting Enzyme 2, HPT: Hypothalamic–Pituitary–Thyroid.

COVID-19 is increasingly recognized for its role in triggering thyroid dysfunction through multifactorial and interrelated mechanisms. The pathogenesis involves both direct effects of the SARS-CoV-2 virus and indirect consequences of the immune and inflammatory responses mounted against the infection. Mechanisms that have been proposed as central to COVID-19-induced thyroid disease are summarized in Figure 5.

#### 4.3.1. Direct Viral Entry and Cytotoxicity

SARS-CoV-2 utilizes the ACE2 receptor, along with the transmembrane protease serine 2 (TMPRSS2), to enter host cells. Thyroid follicular cells express both ACE2 and TMPRSS2 at levels comparable to or even higher than lung cells [59,81], making them direct targets for viral invasion. Autopsy studies have confirmed the presence of SARS-CoV-2 RNA within thyroid tissue, which supports a mechanism of direct cytopathic damage [82]. Viral invasion may lead to cellular apoptosis, disruption of follicular architecture, and release of thyroid antigens, which in turn may provoke inflammation or autoimmunity.

#### 4.3.2. Cytokine-Mediated Suppression of the HPT Axis

The “cytokine storm” associated with severe COVID-19 releases high levels of pro-inflammatory cytokines, particularly IL-6, which can suppress the HPT axis. This suppression may lead to decreased levels of TSH and the impaired peripheral conversion of T4 to T3. Historical data from the 2003 SARS outbreak demonstrated apoptosis of anterior pituitary cells responsible for TSH production, suggesting that coronavirus infections can cause central endocrine disruption [83]. In COVID-19, similar findings have been reported, including low TSH and fT3 levels in severely ill patients [69,84].

#### 4.3.3. Immune-Mediated Autoimmunity

SARS-CoV-2 infection can lead to immune dysregulation that promotes the development of autoimmune thyroid diseases through several interconnected immunopathogenic mechanisms. One such mechanism is molecular mimicry, in which viral antigens share structural similarities with thyroid self-antigens, leading the immune system to mount cross-reactive responses that inadvertently target thyroid tissue. Additionally, the intense inflammatory environment induced by COVID-19 can result in bystander activation, wherein autoreactive lymphocytes are non-specifically activated and contribute to thyroid injury. A further mechanism, known as epitope spreading, may occur when initial immune responses to viral proteins expand to include thyroid-specific antigens, particularly following tissue damage that exposes normally sequestered intracellular components. These pathways collectively help explain the increasing reports of new-onset autoimmune thyroid conditions such as Graves’ disease and Hashimoto’s thyroiditis observed after SARS-CoV-2 infection [21,22].

#### 4.3.4. Endothelial Dysfunction and Vascular Injury

COVID-19 is known to cause widespread endothelial dysfunction, microvascular thrombosis, and coagulopathy. These changes can impair the perfusion of endocrine organs, including the thyroid gland. Impaired blood flow can lead to ischemic injury and contribute to glandular dysfunction, particularly during severe systemic illness [85].

#### 4.3.5. Neuroendocrine Axis Disruption

Beyond peripheral thyroid effects, SARS-CoV-2 may target the hypothalamus and pituitary gland, both of which express ACE2, leading to inflammation, degeneration, or hypophysitis. This may reduce TRH and TSH secretion and contribute to CH. Systemic cytokines may further disrupt hormonal feedback loops, contributing to another layer of HPT axis impairment [76,77].

TFTs, including TSH, fT4, and fT3 within the first few months after recovery, with repeat evaluations over the ensuing six months should occur. Where available, measurement of thyroid autoantibodies, such as anti-TPO, anti-thyroglobulin, and TSH receptor antibodies, may help identify individuals at risk for developing chronic autoimmune thyroid disease. Patients with persistent SCH, recurrent SAT, or autoimmune hyperthyroidism should remain under regular follow-up to monitor for disease progression and to allow timely intervention.

### 4.4. Study Limitations

This systematic review has several limitations that should be considered when interpreting the findings. Most of the included studies were case reports or small case series, with only a few cohort studies. While these detailed reports provide useful insights into prognosis, management, and individual outcomes, they lack the strength and generalizability of larger studies. As a result, it was not possible to draw firm conclusions about the true incidence, prevalence, or severity of thyroid complications after COVID-19.

There was also variability in how thyroid disorders were described. In some cases, hormone levels such as T3 and T4 were reported as high or low without providing TSH levels or autoantibody results, making accurate classification difficult. The duration and quality of follow-up varied widely, from only in-hospital observations to several months post-discharge. This inconsistency made it challenging to assess the long-term course and recovery of thyroid complications. In addition, some reports were unclear about whether patients were recovering, fully recovered, or still recovering with complications, which required interpretation and may have affected some analyses.

Distinguishing complications caused by COVID-19 from those related to pre-existing thyroid conditions was also difficult. Vaccination status was inconsistently reported, often because studies were conducted before vaccine rollout or involved patients ineligible for vaccination, limiting our ability to assess whether vaccination might protect against post-COVID thyroid problems. Finally, although excluding conference abstracts helped ensure data quality and avoid duplication, it may have reduced the overall range of evidence considered.

Despite these limitations, this review provides valuable insight into thyroid complications after COVID-19 across different countries. The findings highlight the need for larger, prospective, multicenter studies with standardized reporting and long-term follow-up to better understand the development and outcomes of these conditions.

## 5. Conclusions

This systematic review reported long-term post-COVID-19 thyroid disorders including SAT, thyrotoxicosis, hyperthyroidism, and hypothyroidism. The evidence suggests that the link between COVID-19 and thyroid disease is multifactorial, with pathogenic mechanisms involving direct viral cytotoxicity, cytokine-driven suppression of the HPT axis, post-viral autoimmune activation, vascular injury, and neuroendocrine disruption.

The clinical course of post-COVID thyroid disorders varied widely. While many cases, particularly those involving thyrotoxicosis or mild SAT, resolved spontaneously, a notable proportion progressed to persistent or recurrent dysfunction, especially in autoimmune conditions such as Hashimoto’s thyroiditis and Graves’ disease. SAT was the most frequently described hyperthyroid-phase disorder, typically emerging within weeks to months after infection and presenting in both painful and painless forms. Thyrotoxicosis was often transient but, in some instances, acted as a precursor to more enduring thyroid disease. Hypothyroidism, including subclinical forms, was frequently a delayed complication and was strongly associated with positive thyroid autoantibodies. CH and NTIS occurred more often in patients with severe or critical COVID-19, reflecting systemic illness and disruption of central endocrine regulation.

Patterns of onset, severity, and recovery were highly heterogeneous, complicated by the inconsistent reporting of thyroid hormone panels, the variable use of autoantibody testing, and the differences in follow-up duration. These limitations, coupled with the predominance of case reports and small series, constrain the ability to establish precise incidence rates or prognostic profiles.

Given these observations, it is advisable that survivors of COVID-19, particularly those with severe illness, prolonged inflammatory responses, or new symptoms suggestive of thyroid dysfunction, undergo comprehensive thyroid function testing, including TSH, free T4, and free T3 within the first few months after recovery, with repeat evaluations over the ensuing six months. Where available, measurement of thyroid autoantibodies, such as anti-TPO, anti-thyroglobulin, and TSH receptor antibodies, may help identify individuals at risk for developing chronic autoimmune thyroid disease. Patients with persistent subclinical hypothyroidism, recurrent SAT, or autoimmune hyperthyroidism should remain under regular follow-up to monitor for disease progression and to allow timely intervention. Thyroid assessment should be incorporated into broader post-COVID and long COVID care pathways, particularly as thyroid dysfunction can contribute to fatigue, mood disturbances, cardiovascular symptoms, and other features commonly reported in this patient population.

In conclusion, COVID-19 is associated with a diverse range of thyroid disorders arising through multiple pathogenic pathways. While many cases are self-limiting, others evolve into chronic disease, making early recognition, structured monitoring, and integrated post-infection care essential to improving patient outcomes.

## Figures and Tables

**Table 1:** Thyroid disorders post-COVID-19 infection.

Type of Thyroid	Number of Patients	Gender	Description	References
SAT	80	19M 61F	5 painless 6 painful 69 NS (61p = 0.082 compared with control)	[27,28,31,32,34,40,42,45,47,48]
Thyrotoxicosis	35	18M 11F 6 NR	17 overt 14 subclinical 4 NS	[29,37,39,41]
Hyperthyroidism	23	1F 22 NR	18 subclinical 5 NS	[29,35,49,51,54]
Graves’ Disease	6	2M 4F	-	[30,32,40,44,50]
High T3	85	24M 19F 42 NR	79 elevated T3 2 T3 toxicosis 1 T3 toxicosis with +anti-TPO and Tg, TSH 3 isolated elevated fT3	[29,37,38,39,52]
High T4	6	NR	6 elevated fT4	[29,37,39,54]
Hypothyroidism	147	3M 2F 142 NR	58 clinical 86 subclinical 2 Hashimoto’s 1 central	[29,32,36,37,39,40,43,46,49,51]
Low T3	1	NR	1 low fT3	[39]
Low T4	3	NR	3 low fT4 (2 with high TSH)	[29,54]
NTIS	22	14 M 6 F 2 NR	20 (low fT3 in COVID rehab group compared with control) *p* = 0.021 1 NTIS 1 low T3, NTIS	[29,37,53]
Other	11	NR	3 high TSH 8 unspecified autoimmune thyroiditis	[33,54]

F: female, fT3: free T3, fT4: free T4, M: male, NR: not reported, NS: not specified, NTIS: Non-Thyroidal Illness Syndrome, SAT: Subacute Thyroiditis, TSH: Thyroid-Stimulating Hormone, TPO: Thyroid Peroxidase, T3: Triiodothyronine, T4: Thyroxine.

## Data Availability

No new data were created or analyzed in this study.

## Supplementary Materials

The following supporting information can be downloaded at https://www.mdpi.com/article/10.3390/microorganisms14030543/s1, Supplementary Table S1: Demographic and clinical data for patients who developed thyroid abnormalities post-COVID-19 infection; Supplementary File S1: The retrieval information file for the systematic review.

## Abbreviations

The following abbreviations are used in this manuscript:

ACE2	Angiotensin-Converting Enzyme 2
CH	Central Hypothyroidism
COVID-19	Coronavirus Disease 2019
CRP	C-Reactive Protein
fT3	Free T3
fT4	Free T4
HR	Hazard Ratio
IL-1β	Interleukin-1 Beta
IL-6	Interleukin-6
IR	Incidence Rate
MIS-A	Multisystem Inflammatory Syndrome in Adults
NAFLD	Non-Alcoholic Fatty Liver Disease
NTIS	Non-Thyroidal Illness Syndrome
PFSAT	Painful SAT
PLSAT	Painless SAT
PRISMA	Preferred Reporting Items for Systematic Reviews and Meta-Analyses
SARS-CoV-2	Severe Acute Respiratory Syndrome Coronavirus 2
SAT	Subacute Thyroiditis
TNF-α	Tumor Necrosis Factor-Alpha
TPO	Thyroid Peroxidase
TSH	Thyroid-Stimulating Hormone
T3	Triiodothyronine
T4	Thyroxine
