# Supplementary material for: Long-Term Thyroid Complications Post-COVID-19: A Systematic Review
Source: Microorganisms. 2026 Feb 27;14(3):543. doi: 10.3390/microorganisms14030543 (PMC13029401; doi:10.3390/microorganisms14030543)
Supplement: Supplementary file 1 [file microorganisms-14-00543-s001.zip › Supplementary Table S1.pdf]

**Supplementary Table S1:** Demographic and clinical data for patients who developed thyroid abnormalities post-COVID-19 infection

| Author | Study Type<br><br>Country | N (total)<br><br>Gender (%M) | Type of thyroid problem | Age Mean ±SE/ Median (IQR) (years)<br><br>Gender (%M) | Comorbidities | COVID-19 Date and duration<br><br>COVID-19 severity | Time of thyroid diagnosis | Blood markers | Treatment<br><br>Vaccination status | Follow up time<br><br>Outcome | QA score |
|---|---|---|---|---|---|---|---|---|---|---|---|
| Lee et al., 2023 [31] | Cross sectional study<br><br>Korea | 407,427<br><br>51.5% M | SAT: 61/407427<br><br>M: 12 (19.7%)<br><br>F: 49 (80.3%)<br><br>p=0.082 (compared with control)<br><br>SAT IR per 1000 persons years<br>95% CI: 16.65 (12.74 - 2.59)<br><br>SAT HR 95% CI:<br>1.93 (1.43–2.59) p<0.001<br><br>Hospitalized:<br>2.00 (1.41 - 2.83)<br>p<0.001<br><br>Non-hospitalized:<br>1.76 (1.01 - 3.06)<br>p=0.045 | 46.3 ± 17.4 | NR | 3 months after COVID<br><br>Hospitalized patients more likely to have SAT | Over 2-year period | NR | NR | Mean follow up 10.8 ± 5.7 months | 8 NOS |
| | | Control: 2,037,135<br><br>51.5% M | SAT: 159/2,037,135<br><br>M: 19 (11.9%)<br><br>F: 140 (88.1%)<br><br>IR per 1000 persons years 95% CI:<br>8.64 (7.35–10.10) | | | | | | | | |

| Author | Study Type<br><br>Country | N (total)<br><br>Gender (%M) | Type of thyroid problem | Age<br>Mean ±SE/ Median (IQR) (years)<br><br>Gender (%M) | Comorbidities | COVID-19 Date and duration<br><br>COVID-19 severity | Time of thyroid diagnosis | Blood markers | Treatment<br><br>Vaccination status | Follow up time<br><br>Outcome | QA score |
|---|---|---|---|---|---|---|---|---|---|---|---|
| Urhan et al., 2022 [38] | Observational cohort study<br><br>Turkey | 43<br><br>55.81% M | T3 is significantly higher than the control group | 44.2 ± 10.7 | HTN: 6<br>HLD: 3<br>T2D: 4 | 2 mild<br>15 moderate<br>22 severe<br>4 critical | Enrolled in study at least 3 months after COVID-19<br><br>No apparent post-COVID symptoms | T3: 3.46 ± 0.38 (p=0.04)<br><br>T4: 1.16 ± 0.12 (p=0.36)<br><br>TSH: 1.83 (1.43-2.67)<br>(p=0.14) | Favipiravir: 20 Pts.<br>Hydroxychloroquine: 33 Pts.<br>Oseltamivir: 11 Pts.<br><br>Glucocorticoid<br>: 7 Pts<br>Tocilizumab: 1 Pt.<br>Low molecular weight heparin: 32 Pts.<br><br>(during treatment of COVID) | 5.6 ±1.3 months | 5 NOS |
| | | Controls: 11<br>54.55% M | | 44.1 ± 12.4 | | | | | | | |
| Ragab, 2022 [48] | Case report<br><br>UAE | 1 M | SAT<br><br>Thyroid pain/tenderness<br><br>Hyperthyroidism symptoms<br><br>Suppressed TSH<br><br>Low thyroid radioactive iodine | 40 | NR | 5 months prior to SAT symptoms<br><br>Asymptomatic | 5 months after recovery from COVID-19 | CRP: 14.6 mg/L<br><br>TSH: 0.89 uIU/ml<br><br>T3: 5.5 pmol/L<br><br>T4: 15.9 pmol/L | Celebrex (200mg) 2x/day<br><br>Acetaminophen/Paracetamol (1000mg) as needed | Total course of 10 weeks (from onset of symptoms to recovery) | 5 Murad et. al |

| Author | Study Type<br><br>Country | N (total)<br><br>Gender (%M) | Type of thyroid problem | Age<br>Mean ±SE/<br>Median (IQR)<br>(years)<br><br>Gender (%M) | Comorbidities | COVID-19<br>Date and duration<br><br>COVID-19 severity | Time of thyroid diagnosis | Blood markers | Treatment<br><br>Vaccination status | Follow up time<br><br>Outcome | QA score |
|---|---|---|---|---|---|---|---|---|---|---|---|
| | | | Elevated erythrocyte sedimentation rate<br><br>Unilateral right neck pain of gradual onset followed by left sided neck pain<br><br>Difficulty swallowing<br><br>Sore throat<br><br>Right lobe of thyroid measured 16.0 cc while left lobe was 6.0 cc | | | | | | | | |
| Garai et al., 2023 [33] | Observational cohort study<br><br>Hungary | 89<br><br>37.1% M | Autoimmune thyroiditis<br><br>6/89 (7%)<br><br>Long COVID | 11.4 ± 3.6 years | NR | NR<br><br>Mild/asymptomatic acute: 94%<br><br>Moderate acute: 6% | 1 - 12+ weeks post infection | TSH (mU/l) Mean: 2.40<br><br>Min: 0.71<br><br>Max: 9.54<br><br>(all patients tested with TSH test) | L-thyroxine therapy<br><br>Unvaccinated | NR | 5 NOS |
| Pizzocaro et al., 2021 [41] | Single-center prospective study<br><br>Italy | 29<br><br>62% M | Thyrotoxicosis resembling SAT<br><br>17 overt 12 subclinical<br><br>TSH increased after 90 days<br><br>Negative for TgAb, TPOAb and TRAb | Median age: 64<br><br>Range: 43-85 | NR | Pts. March 1 - April 1, 2020<br><br>Hospitalized | NR | After a median period of 90 days (30–120)<br><br>Median serum TSH increased from 0.21m U/L (0.05–0.33) to 1.49 mU/L (0.45–6.78) P < 0.001) | NR | Median: 90 days (20-120) after hospital discharge<br><br>40% of patients with COVID related thyrotoxicosis died during hospital stay<br><br>Hormonal level | 6 NOS |

| Author | Study Type<br><br>Country | N (total)<br><br>Gender (%M) | Type of thyroid problem | Age Mean ±SE/ Median (IQR) (years)<br><br>Gender (%M) | Comorbidities | COVID-19 Date and duration<br><br>COVID-19 severity | Time of thyroid diagnosis | Blood markers | Treatment<br><br>Vaccination status | Follow up time<br><br>Outcome | QA score |
|---|---|---|---|---|---|---|---|---|---|---|---|
| | | | | | | | | FT4 decreased (24 cases) from 18.59 pmol/L (12.00–27.66) to 13.00 pmol/L (4.11–17.00)<br><br>P = 0.001 | | increase was detected after recovery | |
| Burekovic et al., 2022 [36] | Retrospective prospective cohort study<br><br>Bosnia and Herzegovina | 248<br><br>7.3% M | Hypothyroidism<br><br>Clinical: 21 and 29 in 2020 and 2021 respectively<br><br>Subclinical: 11 and 7 in 2020 and 2021 respectively | 18 - 70 | NR | COVID-19 cases identified in 2020 and 2021 during the ongoing COVID-19 pandemic | Average time of diagnosis of 2 months after infection for clinical hypothyroidism and 8 weeks for subclinical hypothyroidism | Average TSH in COVID-19 patients: 12.5 (± 2.5-8.5)<br><br>Average TSH in Non-COVID-19 patients: 11.9 (± 2.0-9.5)<br>($p > 0.05$)<br><br>FT3, FT4 and Anti-TPO for COVID-19 vs non-COVID-19: $p > 0.05$ | No significant levothyroxine dosage difference for patients post-COVID-19 infection compared to non-COVID-19 ($p = 0.22$) in 2020 and 2021 | NR | 5 NOS |
| Trinh et al., 2023 [50] | Case report<br><br>Vietnam | 1 F | Graves Disease<br><br>Mild and painless thyromegaly (Goiter)<br><br>Palpitations<br>Shortness of breath when exercising<br><br>US showed mild thyromegaly with a heterogeneous and diffuse hypervascular sonographic appearance | 28 | NR | February 14, 2022<br><br>1 Week<br><br>NR | March 21, 2022<br><br>Developed 4 weeks after COVID-19 | TSH: <0.01 mIU/L<br><br>fT3: 6.18 pmol/L<br><br>fT4: 36.04 pmol/L<br><br>TgAb: 173 IU/mL<br>TRAb:31.7 IU/L<br><br>TPO Ab: >1000 IU/mL | Methimazole (20 mg) | Follow-up 1: 2 months - May 2022<br><br>Follow-up 2: 6 months after follow up 1 - Nov 2022<br><br>Follow-up 3: 3 months after follow up 2 - Feb | 4 Murad et. al |

| Author | Study Type<br><br>Country | N (total)<br><br>Gender (%M) | Type of thyroid problem | Age<br>Mean ±SE/<br>Median (IQR)<br>(years)<br><br>Gender (%M) | Comorbidities | COVID-19<br>Date and duration<br><br>COVID-19 severity | Time of thyroid diagnosis | Blood markers | Treatment<br><br>Vaccination status | Follow up time<br><br>Outcome | QA score |
|---|---|---|---|---|---|---|---|---|---|---|---|
| | | | Decreased TSH<br><br>Elevated fT3<br><br>Elevated fT4<br><br>Elevated thyroglobulin antibodies<br><br>Elevated TRAb<br><br>Elevated TPO Abs | | | | | | | 2023<br><br>Laboratory tests showed improvements after 2 months of treatment | |
| Lui et al., 2021 [37] | Prospective cohort study<br><br>Hongkong/ China | 122<br><br>49.2% M<br><br>Normal TFTs on admission: 102<br><br>Abnormal TFTs on admission: 20 | Subclinical thyrotoxicosis: at baseline and at follow up: 1<br><br>Low fT3 at baseline and at follow up: 1 (suggestive NTIS)<br><br>Low fT3 at baseline then T3 toxicosis at follow up: 1 (persistently positive anti-TPO and anti-Tg but negative anti-TSHR, suggestive of either painless thyroiditis or Graves' disease with negative anti-TSHR)<br><br>Subclinical hypothyroidism at baseline and at follow up: 1 (most likely had pre-existing Hashimoto) | 58 (IQR, 44 - 63) | HTN: 29<br>DM: 19<br>HF: 4<br>Stroke/TIA: 3<br>Malignancy: 3<br>Pulmonary disease: 3 | Admitted July 21- September 21, 2020<br><br>Mild: 99<br>Moderate: 19<br>Severe: 4 | During infection - up to 3 months | Abnormal TFT on admission:<br><br>Low TSH<br><br>Positive anti-TSHR<br><br>Positive anti-TPO<br><br>Positive anti-Tg<br>Low fT3<br><br>Abnormal TFT on reassessment:<br><br>Anti-TPO (35.04 U [IQR 18.76-99.05], P<0.001)<br><br>Anti-Tg (8.71 U [IQR, 6.65 to 15.44], P<0.001)<br><br>Increase in anti-TPO and anti-Tg with interferon | Interferon beta-1b with Ribavirin /and Dexamethasone<br><br>Non was vaccinated | 3 months later<br><br>Normal TFT baseline group: normal TFT = 100<br><br>Isolated elevated T4: 1<br>Subclinical hypothyroidism: 1<br><br>Abnormal baseline TFT: spontaneous resolution: 14<br><br>Subclinical thyrotoxicosis: 1<br>T3<br><br>Thyrotoxicosis: 1<br><br>Low fT3: 1<br><br>Subclinical hypothyroidism: 1 | 7 NOS |

| Author | Study Type<br><br>Country | N (total)<br><br>Gender (%M) | Type of thyroid problem | Age<br>Mean ±SE/<br>Median (IQR)<br>(years)<br><br>Gender (%M) | Comorbidities | COVID-19<br>Date and duration<br><br>COVID-19 severity | Time of thyroid diagnosis | Blood markers | Treatment<br><br>Vaccination status | Follow up time<br><br>Outcome | QA score |
|---|---|---|---|---|---|---|---|---|---|---|---|
| | | | Normal thyroid function test at baseline then Subclinical hypothyroidism at follow up: 1<br><br>Normal thyroid function test at baseline then isolated elevated fT4 at follow up: 1 | | | | | Anti-TSHR (1.0 IU/L [IQR, 0.8 to 1.3], P=0.486)<br><br>fT3 and fT4 within normal range<br><br>Elevated CRP (P=0.033) | | | |
| Mondal et al., 2022 [47] | Retrospective prospective cohort study<br><br>India | 670 COVID19 patients<br><br>160 recovered and followed up | 11/160 (6.9%)<br><br>36.4% M<br><br>Painless SAT: 5<br><br>Painful SAT: 6<br><br>Neck pain<br>Palpitation<br>Tremor<br>Fever<br>Diarrhea | 44.09 ± 16.62 | NR | April 2020 - November 2021<br><br>Moderate: 5<br><br>Severe: 5<br><br>Asymptomatic: 1 | 23.8 days ± 14.8 after recovery | Mean (SD) total T4: 20.51 µg/dL (5.24)<br><br>fT4 levels: 2.27 ng/dL (0.76)<br><br>Mean total T3: 173.49 ng/dL (24.42)<br><br>fT3 levels: 5.8 pg/mL (2.5)<br><br>ESR 70 mm (19.40)<br><br>CRP 134.22 mg/dL (108.74)<br><br>IL-6 levels 77.5 (46.91) | Beta blockers: 11<br><br>NSAIDs: 2<br><br>Glucocorticoids: 4<br><br>Unvaccinated | 3 months and 6 months<br><br>after 6 months: 9 euthyroid<br><br>1 subclinical hypothyroidism<br><br>1 overt hypothyroidism | 6 NOS |

| Author | Study Type<br><br>Country | N (total)<br><br>Gender (%M) | Type of thyroid problem | Age<br>Mean ±SE/ Median (IQR) (years)<br><br>Gender (%M) | Comorbidities | COVID-19 Date and duration<br><br>COVID-19 severity | Time of thyroid diagnosis | Blood markers | Treatment<br><br>Vaccination status | Follow up time<br><br>Outcome | QA score |
|---|---|---|---|---|---|---|---|---|---|---|---|
| Seres et al., 2022 [45] | Case report<br><br>Hungary | 1 F | SAT<br><br>Palpitation<br><br>Acute hyperthyroidism<br><br>Thyroid gland moderately enlarged | 53 | Stage IV sarcoidosis with pulmonary HTN<br><br>End-stage respiratory failure<br><br>Underwent bilateral lung transplant<br><br>*P. aeruginosa* pneumonia<br><br>Lymphopenia | November 25, 2020 - December 10, 2020 | January 14, 2021 after recovery<br><br>(approximately 2 months after infection) | Extreme low thyroglobulin Stimulating hormone<br><br>Elevated antithyroglobulin<br><br>Elevated thyroglobulin levels | Metoprolol<br><br>Heparin<br><br>Methylprednisolone - 250 mg<br><br>Antibiotic Antiviral Antifungal | NR<br><br>Thyroid function normalized after treatment | 5 Murad et. al |
| Whiting et al., 2021 [46] | Case report<br><br>USA | 1 M | Overt hypothyroidism<br><br>Hashimoto's thyroiditis leading to hypothyroidism<br><br>Fatigue<br><br>10-pound weight gain<br><br>Constipation<br><br>Dry skin<br><br>Myalgia | 49 | NR | March 2020 | 6 months after COVID-19 recovery | Initial TSH: 74 mIU/L<br><br>Follow-up TSH: 22.75 mIU/L<br><br>Initial TG: <0.2 ng/mL<br><br>Follow up fT3: 3.12pg/mL<br><br>Follow up fT4: 0.72 ng/dL<br><br>Initial anti-TPO Ab: 626 IU/mL | Synthroid 50 mcg daily | 6 weeks later after initial visit<br><br>Recovered without complications | 6 Murad et. al |
| Houshmand et al., 2021 [34] | Case report<br><br>Iran | 1 M | SAT and active myocarditis<br><br>Persistent atypical chest pain | 25 | NR | COVID-19 duration: 2 weeks | 2 months post recovery | T4: 9.90 mcg/dl<br><br>T3: 2.23 nmol/L<br><br>TSH: 1.3 mIU/ml | β-blocker<br><br>ACE inhibitor<br><br>anti- | Day 70 from diagnosis of COVID-19 | 5 Murad et. al |

| Author | Study Type<br><br>Country | N (total)<br><br>Gender (%M) | Type of thyroid problem | Age<br>Mean ±SE/<br>Median (IQR)<br>(years)<br><br>Gender (%M) | Comorbidities | COVID-19<br>Date and duration<br><br>COVID-19 severity | Time of thyroid diagnosis | Blood markers | Treatment<br><br>Vaccination status | Follow up time<br><br>Outcome | QA score |
|---|---|---|---|---|---|---|---|---|---|---|---|
| | | | Dyspnea<br><br>Sore throat<br><br>Neck swelling and thyroid tenderness | | | | | CRP - 9.9 mg/dL | inflammatory agent | | |
| Abusaiba et al., 2021 [42] | Case report<br><br>Iraq | 1 M | SAT<br><br>Red neck<br><br>Swollen and tender hyperemic tonsils<br><br>Fever and tachycardia<br><br>Sore throat with pain radiating to lower jaw | 43 | None | NR | 6 weeks after tested (+) for COVID-19 | TSH/thyrotropin ratio: 0.95uIU/ml<br><br>T4: 95.7 umol/ml<br><br>T3: 1.5umol/ml<br><br>CRP: 35 mg/dL<br>ESR: 65 mm/h | NR | 6 weeks after recovery | 5 Murad et. al |
| Alphan et al., 2023 [49] | Observational cohort study<br><br>Turkey | 163<br><br>54.6% M | At 6 months follow up:<br><br>Euthyroid: 117<br><br>Subclinical hyperthyroidism: 2<br><br>Subclinical hypothyroidism: 5 | Age: 58 (29–95)<br><br>52.2% M | DM: 30<br>HTN: 39<br>COPD: 16<br>Obesity: 40<br>CAD: 16 | Mild-Moderate: 31<br><br>Severe: 61 | 56.4% had thyroid dysfunction admission<br><br>5.6% still showed dysfunction after 6 months | Baseline lab values:<br><br>TSH (mIU/L): 0.85 (0.04-8.10) ($p<0.001$)<br><br>fT4 (ng/dL): 1.11 (0.20-2.48) ($p=0.017$)<br><br>fT3 (ng/dL): 2.09 (0.59-2.81) ($p<0.001$)<br><br>Anti-Tg (U/mL): 3.0 (0-258) ($p=0.874$)<br><br>Anti-TPO (U/mL):<br>31.1 (0-1300) ($p=0.038$) | NR | 6 months<br><br>124 patients survived (76.1%) and 39 died (23.9%) | 8 NOS |

| Author | Study Type<br><br>Country | N (total)<br><br>Gender (%M) | Type of thyroid problem | Age<br>Mean ±SE/<br>Median (IQR)<br>(years)<br><br>Gender (%M) | Comorbidities | COVID-19<br>Date and duration<br><br>COVID-19 severity | Time of thyroid diagnosis | Blood markers | Treatment<br><br>Vaccination status | Follow up time<br><br>Outcome | QA score |
|---|---|---|---|---|---|---|---|---|---|---|---|
| | | | | | | | | Post discharge 6th Month lab values for all survivors (n=124) (mean ± SD):<br><br>TSH (mIU/L): 1.97 ±1.30 ($p<0.001$)<br><br>fT3 (ng/dL): 3.05±0.38 ($p<0.001$)<br><br>fT4 (ng/dL): 1.10±0.18 ($p=0.017$)<br><br>Anti-Tg (U/mL): 22.5 ± 63.2 ($p=0.874$)<br><br>Anti-TPO (U/mL): 120.4 ± 291.7 ($p=0.038$) | | | |
| Elhadd et al., 2022 [32] | Case series<br><br>Qatar | 10<br><br>30% M | 6/10<br><br>(4 Pts had history of thyroid problems) | Mean: 37.4±3.55<br><br>Median: 36.5 (14.5) | | | | | | | |
| | | | Pt. 1: Hypothyroidism.<br><br>Weight gain | 49<br>M | HTN<br>Asthma<br>Prediabetes<br>Gout | 24/11/20<br><br>NR | 6 weeks after recovery | Initially: TSH: 33.49 mu/L<br><br>T4: 8 pmol/L<br><br>6 weeks later: | Levothyroxine | 2 months, then 6 weeks and then 6 weeks | 4 Murad et. al |

| Author | Study Type<br><br>Country | N (total)<br><br>Gender (%M) | Type of thyroid problem | Age Mean ±SE/ Median (IQR) (years)<br><br>Gender (%M) | Comorbidities | COVID-19 Date and duration<br><br>COVID-19 severity | Time of thyroid diagnosis | Blood markers | Treatment<br><br>Vaccination status | Follow up time<br><br>Outcome | QA score |
|---|---|---|---|---|---|---|---|---|---|---|---|
| | | | | | | | | TSH: 34.22 mu/L<br><br>T4: 7.4 pmol/L<br><br>Referred to endo clinic TSH: 49 mu/L<br><br>FT: 11 pmol/L<br><br>Anti-TPO Ab: 423 IU/mL<br><br>TRAb (-)<br><br>6 more weeks: TSH: 61 mIU/L<br><br>FT: 4 pmol/L | | | |
| | | | Pt. 2: Grave's disease Thyroid storm later due to stopping antithyroid drugs<br><br>Persistent tachycardia<br><br>Abdominal pain<br><br>Vomiting<br><br>HTN<br><br>Tachycardia and small goiter | 40<br>F | Asthma | NR<br><br>Mild/moderate | 2 weeks<br><br>Thyroid storm at 8 months | TSH <0.01 mIU/L<br><br>fT3: 30.8 pmol/L<br><br>fT4: 83.3 pmol/L<br><br>Anti-TPO Abs: 494 IU/mL<br><br>TRAb: 8.7 IU/L<br><br>8 months: TSH:<0.01mIU/L<br><br>fT3: 32.9 pmol/L<br><br>fT4: >100 pmol/L<br><br>TRAb: 7.7u/L | Carbimazole & metoprolol | 8 months | |
| | | | Pt.3 | 40 | NR | NR | 2-3 months | TSH: <0.01mIU/L | Carbimazole & propranolol | 2 months | |

| Author | Study Type<br><br>Country | N (total)<br><br>Gender (%M) | Type of thyroid problem | Age Mean ±SE/ Median (IQR) (years)<br><br>Gender (%M) | Comorbidities | COVID-19 Date and duration<br><br>COVID-19 severity | Time of thyroid diagnosis | Blood markers | Treatment<br><br>Vaccination status | Follow up time<br><br>Outcome | QA score |
|---|---|---|---|---|---|---|---|---|---|---|---|
| | | | Grave's disease<br><br>Weight loss and anxiety | M | | | | fT4:100 pmol/L | | TFTs normalized | |
| | | | Pt. 4:<br>Thyroiditis<br><br>Weight loss and palpitations | 14<br><br>F | NR | NR<br><br>Mild | 6 weeks after COVID-19 | TSH: <0.02mU/L<br>T4: 35 pmol/L.<br><br>5 weeks:<br>TSH: 5.6 mIU/L<br>fT4: 25 pmol/L<br><br>2 months:<br>TSH: 7.5 mIU/L<br>fT4: 17 pmol/L | Propranolol | Referral in Nov 2020<br><br>5 weeks later<br><br>March 2021:Euthyroidis m | |
| | | | Pt. 5:<br>Grave's disease<br><br>Weight loss palpitations<br><br>Increased sweating and shakiness | | NR | October 2020.<br><br>Mild | 4 weeks after infection | TSH: <0.01mIU/L<br><br>fT4: 46 pmol/L<br><br>TRAb: 8.7 IU/L<br><br>Anti-TPO Ab: 73IU/L | Carbimazole & propranolol | NR<br><br>Euthyroid | |
| | | | Pt. 6:<br>Central hypothyroidism<br><br>Aches pain and joint discomfort | 33<br><br>F | NR | 19 June, 21 | 2 months | August 2021:<br><br>T4: 4.1 pmol/L<br><br>TSH: 6.5 mIU/L | Levothyroxin e | Serial TFTs till December 2021<br><br>Persistent central hypothyroidism: replacement therapy indicated | |
| DeGiglio et al., 2023 [44] | Case report<br><br>Italy | 1 M | Graves Disease<br><br>US showing heterogeneous thyroid echotexture and hypervascularisation | 75 | Mild diplopia | November 2020<br><br>Recovered at 3 weeks from diagnosis | February 2021<br><br>About 6 weeks after recovery | Suppressed TSH: 0.014 uUI/ml<br><br>Increased fT4: 5.37 ng/dL<br><br>+ Anti-TSH receptor Ab: 27 IU/L | Tapazole therapy | 6 months<br><br>Stable recovery of ocular motility | 5 Murad et. al |

| Author | Study Type<br><br>Country | N (total)<br><br>Gender (%M) | Type of thyroid problem | Age<br>Mean ±SE/ Median (IQR) (years)<br><br>Gender (%M) | Comorbidities | COVID-19 Date and duration<br><br>COVID-19 severity | Time of thyroid diagnosis | Blood markers | Treatment<br><br>Vaccination status | Follow up time<br><br>Outcome | QA score |
|---|---|---|---|---|---|---|---|---|---|---|---|
| Croce et al., 2023 [53] | Case-control study<br><br>Italy | Control (post-COVID-19 non-rehabilitation): 19<br><br>63.2% M | NTIS<br>19<br><br>Reduced fT3 & fT4 | 60.5 ± 12.3 | CIRS-CI: 1.84 ± 1.46<br><br>CIRS-SI: 1.34 ± 0.20 | March 13, 2020 - July 31, 2021<br><br>Acute illness | 4 weeks after recovery | Median (IQR):<br><br>fT3<br>Initial:<br>2.89 (2.65-3.07) pg/ml<br>(p=0.672 vs follow up)<br><br>Follow-up:<br>2.78 (2.62-2.98) pg/ml<br><br>fT4<br>Initial:<br>0.92 (0.86-1.07) ng/dl<br>(p=0.286 vs follow up)<br><br>Follow-up:<br>0.95 (0.89-1.04) ng/dl<br><br>TSH<br>Initial:<br>1.35 (0.99-1.69) U/L<br>(p=0.601 vs follow up)<br><br>Follow-up:<br>1.19 (1.02-1.81) U/L | NR | 3 months | 7 NOS |
| | | Post-COVID-19 (rehabilitation): 20 | NTIS<br>20 | 67.2 ± 8.0 | CIRS-CI:<br>3.40 ± 1.31<br>p=0.001<br><br>CIRS-SI: | | | fT3<br>Initial:<br>2.54 (2.32-2.82) pg/ml | | | |

| Author | Study Type<br><br>Country | N (total)<br><br>Gender (%M) | Type of thyroid problem | Age<br>Mean ±SE/<br>Median (IQR)<br>(years)<br><br>Gender (%M) | Comorbidities | COVID-19<br>Date and duration<br><br>COVID-19 severity | Time of thyroid diagnosis | Blood markers | Treatment<br><br>Vaccination status | Follow up time<br><br>Outcome | QA score |
|---|---|---|---|---|---|---|---|---|---|---|---|
| | | 4 weeks of respiratory rehabilitation after recovery from COVID-19<br><br>70% M | | | 1.60 ± 0.24<br>p<0.001 | | | (p=0.021 vs control, p=0.007 vs follow up)<br><br>Follow-up: 2.89 (2.65-3.07) pg/ml<br><br>(p=0.627 vs control)<br><br>fT4<br>Initial: 0.86 (0.74-0.94) ng/dl<br>(p=0.134 vs control, p=0.243 vs follow up)<br><br>Follow-up:<br>0.93 (0.75-0.97) ng/dl<br>(p=0.113 vs control)<br><br>TSH<br>Initial: 1.48 (1.12-2.99) U/L<br>(p=0.189 vs control, p=0.502 vs follow up)<br><br>Follow-up:<br>1.88 (1.34-2.63) U/L<br>(p=0.079 vs control) | | | |
| Semenova et al., 2023 [54] | longitudinal case-control study | 57 F | Euthyroidism: 43 | 58 ± 6.33<br><br>100% F | NR | June 2020 – March, 2021 | During active infection | T4: 16.7 (14.2-18.5) pmol/l | NR | 12 months from recovery | 6 NOS |

| Author | Study Type<br><br>Country | N (total)<br><br>Gender (%M) | Type of thyroid problem | Age<br>Mean ±SE/<br>Median (IQR)<br>(years)<br><br>Gender (%M) | Comorbidities | COVID-19<br>Date and duration<br><br>COVID-19 severity | Time of thyroid diagnosis | Blood markers | Treatment<br><br>Vaccination status | Follow up time<br><br>Outcome | QA score |
|---|---|---|---|---|---|---|---|---|---|---|---|
| | Russia | (COVID-19 with pneumonia)<br><br>15 (control) asymptomatic without IgG<br><br>13 (separate group) asymptomatic with IgG | Subclinical hyperthyroidism: 7<br><br>High TSH: 3<br><br>High fT4: 2<br><br>Low fT4: 1<br><br>Low fT4 and high TSH: 1 | | | Moderate | | CRP: 11.7 (6.8-13.1) pg/ml)<br><br>TNF-alpha: 1.7 (1.1-2.5) pg/ml<br><br>Unvaccinated | | Persistence of elevated T4 levels | |
| Yanachkova et al., 2023 [51] | Observational prospective cohort study<br><br>Bulgaria | 113<br><br>31% M | 2 months after COVID-19<br><br>Euthyroid: 44/113 (38.9%)<br><br>Subclinical hypothyroidism: 54/113 (47.8%)<br><br>Overt Hypothyroidism: 6/113 (5.3%)<br><br>Subclinical hyperthyroidism: 9/113 (8%)<br><br>Negative for both TPO Ab and TgAb: 47/113 (41.6%)<br><br>Positive for both TPO Ab and TgAb = 34/113 (30.1%)<br><br>TgAb Positive 55/113 (48.7%) | 43.0 ± 5 | NR | NR<br><br>Mild to moderate | 2 months after infection | TSH: 5.73 (4.91-6.53) mIU/L (p=0.008)<br><br>T4: 13.81 (12.84-14.5) pmol/L (p=0.014) | Levothyroxine therapy<br><br>Heparin or antiplatelet agents<br><br>Glucorticoids | 3 months after infection<br><br>Euthyroid: 81<br>Subclinical hypothyroidism: 32 | 6 NOS |

| Author | Study Type<br><br>Country | N (total)<br><br>Gender (%M) | Type of thyroid problem | Age<br>Mean ±SE/ Median (IQR) (years)<br><br>Gender (%M) | Comorbidities | COVID-19 Date and duration<br><br>COVID-19 severity | Time of thyroid diagnosis | Blood markers | Treatment<br><br>Vaccination status | Follow up time<br><br>Outcome | QA score |
|---|---|---|---|---|---|---|---|---|---|---|---|
| | | | (p = 0.03 vs TgAb negative group)<br><br>TPOAb Positive: 45/113 (39.8%)<br><br>3 months after COVID-19:<br>Euthyroid : 81/113 (71.7%)<br><br>Subclinical hypothyroidism: 32/113 (28.3%) | | | | | | | | |
| Feghali et al., 2021 [40] | Case reports<br><br>USA | 3 F | Pt. 1<br>Hashimoto<br><br>Thyroiditis<br><br>Throat pain<br><br>Myalgia<br><br>Fatigue<br><br>Diarrhea<br><br>Thyroid enlargement<br><br>Dry skin<br><br>Hair loss<br><br>Worsening depression | 38<br><br>F | NR | May 4, 2020 | 6 weeks from infection,<br><br>1 week after recovery | TSH: 136 mIU/L<br><br>fT4: 0.2 ng/dL<br><br>TGAb: >1000IU/mL<br><br>TPO: >900 IU/mL | Thyroid hormone replacement | Improved over 4 weeks | 6 Murad et. al |
| | | | Pt. 2<br><br>Graves' disease<br><br>Chills | 33<br><br>F | NR | April 28, 2020 | 7 weeks from infection | TSH:<0.01mIU/L<br><br>fT4: 2.1 ng/dl<br><br>Thyroglobulin: 8.8 ng/ml | Propranolol<br><br>Methimazole | Symptoms relief with resolution of palpitations in 2 weeks | |

| Author | Study Type<br><br>Country | N (total)<br><br>Gender (%M) | Type of thyroid problem | Age<br>Mean ±SE/<br>Median (IQR)<br>(years)<br><br>Gender (%M) | Comorbidities | COVID-19<br>Date and duration<br><br>COVID-19 severity | Time of thyroid diagnosis | Blood markers | Treatment<br><br>Vaccination status | Follow up time<br><br>Outcome | QA score |
|---|---|---|---|---|---|---|---|---|---|---|---|
| | | | Fever<br><br>Diarrhea<br><br>Fatigue<br><br>Headache<br><br>Sinus<br><br>Pain<br><br>Dry cough | | | | | TGAb: 4 IU/mL<br><br>TRAb: <1IU/L<br><br>TSI: 309% | | | |
| | | | Pt. 3<br><br>SAT<br><br>Persistent palpitations and insomnia | 41<br><br>F | NR | September 15th, 2020 | 6 weeks from infection | TSH: 0.01 mIU/L<br><br>fT: 1.9 ng/dL<br><br>Thyroglobulin: 3IU/mL<br><br>TPO: 69 IU/mL<br><br>TRAb: 1IU/L<br><br>TSI: 89% | Thyroid hormone supplementation with levothyroxine (after follow-up) | Developed hypothyroidism 3 weeks after first visit and then started on meds | |
| Al-Shammaa et al., 2022 [27] | Case report<br><br>Iraq | 1 F | SAT<br><br>Palpitation<br><br>Sweating<br><br>Agitation<br><br>Neck pain<br>Increased pulse<br><br>Tremors<br><br>Malaise | 53 | None | November 2020<br><br>9 days<br><br>Mild | 10 days after resolution of COVID-19 infection | T3: 3.69 nmol/L<br><br>T4: 234.2 nmol/L<br><br>Free T3: 13.8 pmol/L<br>Free T4: 31.6 pmol/L<br><br>TSH: 0.021 mIU/L | Beta blockers<br><br>Low dose of analgesia | 3 Months<br><br>Thyroid function returned to normal values after three months of therapy | 6 Murad et. al |

| Author | Study Type<br><br>Country | N (total)<br><br>Gender (%M) | Type of thyroid problem | Age Mean ±SE/ Median (IQR) (years)<br><br>Gender (%M) | Comorbidities | COVID-19 Date and duration<br><br>COVID-19 severity | Time of thyroid diagnosis | Blood markers | Treatment<br><br>Vaccination status | Follow up time<br><br>Outcome | QA score |
|---|---|---|---|---|---|---|---|---|---|---|---|
| | | | Neck pain<br><br>Fatigue | | | | | Anti-thyroperoxidase Ab: 13.7 U/ml<br><br>Anti-thyroglobulin Ab: 26.8 U/ml<br><br>TSH receptor Ab: 1.24 IU/L | | | |
| Bhatt et al., 2023 [43] | Case report<br><br>India | 1 M | Post-MIS-A Thyroiditis leading to hypothyroidism | Mid-thirties | None | NR | After recovery from COVID-19 | TSH: 37.25 µIU/mL<br><br>Anti-TPO antibodies: 191.69 IU/mL<br><br>Unvaccinated | Intravenous furosemide<br><br>Supplemental oxygen<br><br>Aspirin (75 mg once daily)<br><br>Clopidogrel (75 mg once daily)<br><br>Digoxin (0.25 mg once daily for 5 days a week)<br><br>sacubitril-valsartan (50mg twice daily)<br><br>rivaroxaban (10 mg once a | 6 months: euthyroid symptomatically better | 4 Murad et. al |

| Author | Study Type<br><br>Country | N (total)<br><br>Gender (%M) | Type of thyroid problem | Age<br>Mean ±SE/<br>Median (IQR)<br>(years)<br><br>Gender (%M) | Comorbidities | COVID-19<br>Date and duration<br><br>COVID-19 severity | Time of thyroid diagnosis | Blood markers | Treatment<br><br>Vaccination status | Follow up time<br><br>Outcome | QA score |
|---|---|---|---|---|---|---|---|---|---|---|---|
| | | | | | | | | | day)<br><br>L-thyroxine (100 mcg daily) | | |
| Lui et al., 2021 [39] | Prospective cohort study<br><br>China | 204<br><br>46.6% M<br><br>172 Symptomatic in acute COVID-19<br><br>47.7% M<br><br>32 Asymptomatic in acute COVID-19<br><br>40.6% M | Subclinical thyrotoxicosis at baseline and at follow up: 3<br><br>Low fT3 at baseline and at follow up: 1<br><br>Low fT3 at baseline then T3 toxicosis at follow up: 1<br><br>Subclinical hypothyroidism at baseline and at follow up: 3<br><br>Normal thyroid function test at baseline then subclinical hypothyroidism at follow up: 1<br><br>Normal thyroid function test at baseline then isolated elevated fT4 at follow up: 1<br><br>Normal thyroid function test at baseline then isolated elevated fT3 at follow up: 1 | Total: 55.0 (44.3-63.0)<br><br>Symptomatic:<br>56.0 (45.0-63.0)<br>47.7% M<br><br>Asymptomatic<br>53.5 (32.0-64.5)<br>40.6% M | HTN: 23.0%<br>DM:13.7%<br>Obesity:7.4% | July 21 - December 21, 2020<br><br>Mild: 147<br>Moderate 49<br>Severa: 8 | 89 days (IQR, 69-99) | Symptomatic:<br><br>TSH (mIU/L): 1.45 (0.96-2.00) (P= 0.080)<br><br>fT4 (pmol/L): 17 (15-19) (p=0.470)<br><br>fT3 (pmol/L): 4.7 (4.3-5.0) (p=0.002)<br><br>Anti TPO positivity: 20.1% (p=0.014)<br><br>Anti Tg positive: 11.5% (p=0.999)<br><br>Asymptomatic<br>TSH (mIU/L): 1.40 (0.88-1.78)<br><br>fT4 (pmol/L): 18 pmol/L(16-19)<br><br>fT3 (pmol)/L: 4.9 (4.4-5.2) | NR | NR | 8 NOS |

| Author | Study Type<br><br>Country | N (total)<br><br>Gender (%M) | Type of thyroid problem | Age Mean ±SE/ Median (IQR) (years)<br><br>Gender (%M) | Comorbidities | COVID-19 Date and duration<br><br>COVID-19 severity | Time of thyroid diagnosis | Blood markers | Treatment<br><br>Vaccination status | Follow up time<br><br>Outcome | QA score |
|---|---|---|---|---|---|---|---|---|---|---|---|
| | | | | | | | | Anti TPO positivity: 43.5%<br><br>Anti-Tg positive: 8.7% | | | |
| Mehmood et al. 2020 [28] | Case report<br><br>USA | 1 F | SAT<br><br>Difficulty swallowing<br><br>Fever<br><br>Exertional tachycardia<br><br>Weight loss<br><br>Shortness of breadth<br><br>Heterogeneously enlarged thyroid gland<br><br>Fine bilateral hand tremors<br><br>Palpable left thyroid lobe compared to right | 29 | NR | NR<br><br>Recovered after a 5 day course of azithromycin and hydroxychloroquine<br><br>Mild | 7 weeks post COVID-19 | T4: 4.4 ng/L<br><br>T3: 372 ng/L<br><br>TSH: 0.01 mu/L | Discharged home with: Prednisone (20 mg)<br><br>Atenolol (25 mg)<br><br>After 3 days follow up: Prednisone (40 mg)<br><br>Atenolol (50 mg) | 10 weeks<br><br>Asymptomatic and normal thyroid function tests after 10 weeks | 6 Murad et. al |
| Lui et al., 2023 [29] | Prospective follow up study<br><br>Hong Kong | 250<br><br>50.4% M | SAT at baseline and at 3 months<br><br>4/236 (1.7%)<br><br>**A**t 6 months: 2/163 (1 suggested Graves' disease and 1 possible toxic thyroid nodules)<br><br>Low fT3 at baseline and at 3 months: 1/236 suggesting nonthyroidal illness syndrome (NTIS) | 52.7 ± 15.3 | DM: 36 (14.4%)<br><br>HTN: 60 (24.0%)<br><br>IHD/CHF: 11 (4.4%)<br><br>Stroke/TIA: 5 (2.0%)<br><br>Malignancy: 16 (6.4%) | 21 July 2020 - 20 May 2021<br><br>Mild: 168 (67.2%)<br><br>Moderate: 70 (28.0%)<br><br>Severe: 12 (4.8%) | 3-6 months | TSH: 0.35–4.8 mIU/L<br><br>fT4: 12–23 pmol/L<br><br>fT3: 3.2–6.5 pmol/L | Interferon beta-1b<br><br>Unvaccinated | 3-6 months | 6 NOS |

| Author | Study Type<br><br>Country | N (total)<br><br>Gender (%M) | Type of thyroid problem | Age<br>Mean ±SE/<br>Median (IQR)<br>(years)<br><br>Gender (%M) | Comorbidities | COVID-19<br>Date and duration<br><br>COVID-19 severity | Time of thyroid diagnosis | Blood markers | Treatment<br><br>Vaccination status | Follow up time<br><br>Outcome | QA score |
|---|---|---|---|---|---|---|---|---|---|---|---|
| | | | T3 toxicosis at baseline and at 3 months: 1/236 normalized after 6 month suggesting painless thyroiditis<br><br>Subclinical hypothyroidism at baseline and at 3 months: 3/236<br>6 months: 2/163<br><br>Normal thyroiditis at baseline then:<br>Subclinical hypothyroidism at 3 months: 1/236, 6 months: 1/163<br><br>Isolated elevated fT4 at 3 months: 2/236<br><br>Isolated elevated fT3 at 3 months: 1/236<br><br>Isolated low fT4 at 3 months: 1/236<br><br>Normal thyroiditis at baseline and at 3 months follow up then:<br><br>Subclinical thyrotoxicosis at 6 months: 2/147<br><br>Isolated elevated fT3 at 6 months: 1/147 | | Pulmonary disease: 13 (5.2%) | | | | | | |

| Author | Study Type<br><br>Country | N (total)<br><br>Gender (%M) | Type of thyroid problem | Age<br>Mean ±SE/ Median (IQR) (years)<br><br>Gender (%M) | Comorbidities | COVID-19 Date and duration<br><br>COVID-19 severity | Time of thyroid diagnosis | Blood markers | Treatment<br><br>Vaccination status | Follow up time<br><br>Outcome | QA score |
|---|---|---|---|---|---|---|---|---|---|---|---|
| | | | Subclinical hypothyroidism at 6 months: 1/147 | | | | | | | | |
| Malik et al., 2021 [52] | Retrospective cross-sectional study<br><br>Pakistan | 76<br><br>60.5% M<br><br>48: COVID-19<br><br>28: Non-COVID pneumonia | 36/48 (75%)<br><br>Elevated TT3<br><br>TT3 levels were raised significantly 3 months after infection in COVID-19 than non-COVID-19 patients with little to no discernable effect on TT4 levels: 48 | 51 ± 19.30 | No clinically significant ones | NR<br><br>Moderate: 22 (45.8%)<br><br>Severe: 9 (18.8%)<br><br>Critical: 17(35.4%) | Detected on day one before initiation of treatment | IL6: 14.16 ± 16.58 (pg/mL)<br><br>TSH: 1.11 ± 1.15 (mIU/L)<br><br>T3: 89.98 ± 35.15 (ng/dL)<br><br>T4: 6.79 ± 2.37 (mcg/dL) | Corticosteroids for severe COVID-19 Pts. | 3 months<br><br>Thyroid hormones returned to normal after recovery | 6 NOS6 |
| Qureshi et al., 2021 [30] | Case report<br><br>USA | 1 M | Hyperthyroidism from autoimmune thyroid disease<br><br>Mild exophthalmos<br><br>Palpable thyroid<br><br>Increased appetite<br><br>Weight loss<br><br>Fatigue<br><br>Heat intolerance<br><br>2 month follow up: Palpitations<br><br>Difficulty sleeping<br><br>Heat intolerance<br><br>4 month Follow up: | 13 | None | NR<br><br>Mild | 8 weeks post COVID-19 infection | T4: 1.2 ng/dl<br><br>TSH: <0.01 uIU/mL | Methimazole (thionamide therapy)10mg once daily<br><br>2 month follow up: Propranolol 10 mg twice daily | 2 months and 4 months | 6 Murad et. al |

| Author | Study Type<br><br>Country | N (total)<br><br>Gender (%M) | Type of thyroid problem | Age<br>Mean ±SE/<br>Median (IQR)<br>(years)<br><br>Gender (%M) | Comorbidities | COVID-19<br>Date and duration<br><br>COVID-19 severity | Time of thyroid diagnosis | Blood markers | Treatment<br><br>Vaccination status | Follow up time<br><br>Outcome | QA score |
|---|---|---|---|---|---|---|---|---|---|---|---|
| | | | Insomnia<br><br>Anxiety<br><br>Depression<br><br>Poor concentration<br><br>Palpitations<br><br>Difficulty sleeping<br><br>Heat intolerance | | | | | | | | |
| Ilyaev et al., 2023 [35] | Case report<br><br>USA | 1 F | COVID-19 induced hyperthyroidism<br><br>Neck pain<br><br>Difficulty swallowing<br><br>Tremors<br><br>Shaking<br><br>Sweating<br><br>Weight loss<br><br>Anxiety<br><br>Fever | 30 | None | 2 weeks | Diagnosed ost-COVID-19 recovery | Elevated CRP<br><br>Suppressed TSH<br><br>Elevated T4 | Prednisone (to decrease swelling and inflammation of thyroid gland) | 11 day post initial diagnosis<br><br>Not recovered from hyperthyroidism until the time of writing this paper | 4 Murad et. al |

Ab: Antibody, CAD: Coronary Artery Disease, COPD: Chronic Obstructive Pulmonary Disease, CRP: C-Reactive Protein, CIRS-SI: Cumulative Illness Rating Scale Severity Index, DM: Diabetes Mellitus, ESR: Erythrocyte Sedimentation Rate, HF: Heart Failure, HTN: Hypertension, HLD: hyperlipidemia, IHD: Ischemia Heart Disease, IL: Interleukin, Pt.: Patient, T3: triiodothyronine, SAT: Subacute Thyroiditis, T2D: Type 2 diabetes, TIA: Transient Ischemia Attack, TSH: Thyroid Stimulating Hormone, TgAb: Thyroglobulin Antibody, TRAb: Thyrotropin Receptor Antibody, TPO: Thyroid Peroxidase, TSI: Thyroid Stimulating Immunoglobulin, TNF: Tumor Necrosis Factor, T4: Thyroxine, fT3: free T3, fT4: free T4, US: Ultra Sound.
